# Association between door-to-needle time and outcomes in acute ischemic stroke patients treated with intravenous thrombolysis plus mechanical thrombectomy: Analysis from the Italian Registry of Endovascular Treatment in Acute Stroke (IRETAS)

**DOI:** 10.1093/esj/23969873251368720

**Published:** 2026-01-01

**Authors:** Fabrizio Sallustio, Alfredo Paolo Mascolo, Federico Marrama, Marina Diomedi, Giordano Lacidogna, Federica D’Agostino, Fana Alemseged, Valerio Da Ros, Federico Sabuzi, Enrico Fainardi, Ilaria Casetta, Stefano Vallone, Guido Bigliardi, Luca Allegretti, Elena Coco, Elvis Lafe, Marco Longoni, Vittorio Semeraro, Giovanni Boero, Benedetto Petralia, Manuel Cappellari, Ettore Nicolini, Antonio Ciacciarelli, Rosa Napoletano, Andrea Boghi, Andrea Naldi, Andrea Saletti, Alessandro De Vito, Sergio Lucio Vinci, Ludovica Ferraù, Domenico Sergio Zimatore, Marco Petruzzellis, Mauro Bergui, Giovanni Bosco, Ivan Gallesio, Delfina Ferrandi, Mirco Cosottini, Nicola Giannini, Alessio Comai, Elisa Dall’Ora, Giovanni Barchetti, Marcella Caggiula, Nicola Cavasin, Adriana Critelli, Marco Perri, Federica De Santis, Simone Galluzzo, Andrea Zini, Simone Zilahi De Gyurgyokai, Nicola Loizzo, Roberto Menozzi, Alessandro Pezzini, Massimo Sponza, Giovanni Merlino, Marco Filizzolo, Marina Mannino, Giuseppe Carità, Monia Russo, Massimiliano Allegritti, Stefano Caproni, Michele Besana, Alessia Giossi, Samuele Cioni, Rossana Tassi, Gianluca Galvano, Eleonora Saracco, Nicola Limbucci, Edoardo Puglielli, Alfonsina Casalena, Salvatore Mangiafico, Danilo Toni

**Affiliations:** Unità Di Trattamento Neurovascolare, Ospedale Dei Castelli-ASLRoma6, Rome, Italy; Comprehensive Stroke Center, Department of Systems Medicine, University of Tor Vergata, Rome, Italy; Comprehensive Stroke Center, Department of Systems Medicine, University of Tor Vergata, Rome, Italy; Comprehensive Stroke Center, Department of Systems Medicine, University of Tor Vergata, Rome, Italy; Comprehensive Stroke Center, Department of Systems Medicine, University of Tor Vergata, Rome, Italy; Comprehensive Stroke Center, Department of Systems Medicine, University of Tor Vergata, Rome, Italy; Royal Melbourne Hospital, Melbourne, Australia; Interventional Radiology Unit, Department of Biomedicine and Prevention, University of Tor Vergata, Rome, Italy; Interventional Radiology Unit, Department of Biomedicine and Prevention, University of Tor Vergata, Rome, Italy; Dipartimento di Scienze Biomediche, Sperimentali e Cliniche, Neuroradiologia, Università degli Studi di Firenze, Ospedale Universitario Careggi, Firenze, Italy; Neurology Unit, University of Ferrara, Ferrara, Italy; UO Neuroradiologia, AOU Modena, Modena, Italy; UO Neurologia - Stroke Unit, AOU Modena, Modena, Italy; SC Neuroradiologia Osp. Santa Corona, Pietra Ligure Asl2 Liguria, Italy; SC Neurologia e Centro Ictus, Ospedale Santa Corona, Pietra Ligure Asl2 Liguria, Italy; UO Neuroradiologia Interventistica Romagna, Cesena, Italy; UO Neurologia e Stroke Unit, Cesena, AUSL Romagna, Italy; SC Neuroradiologia Ospedale “SS., Annunziata” Taranto, Italy; S. C. Neurologia e Stroke Unit - Ospedale “SS., Annunziata” Taranto, Italy; UOC Neuroradiologia AOUI Verona, Italy; Stroke Unit - Azienda Ospedaliera Universitaria Integrata Verona, Italy; UOSD UTN - Policlinico Umberto I - Università La Sapienza Roma, Italy; UOSD UTN - Policlinico Umberto I - Università La Sapienza Roma, Italy; UOC Radiologia Interventistica e Neurointerventistica AOU S. Giovanni di Dio e Ruggi d’Aragona, Salerno, Italy; UOSD Stroke Unit AOU S. Giovanni di Dio e Ruggi d’Aragona, Salerno, Italy; Neuroradiologia, Ospedale San Giovanni Bosco, Torino, Italy; Neurology Unit, San Giovanni Bosco Hospital, Turin, Italy; UOC Neuroradiologia - AOU, Ferrara, Italy; UO Neurologia - Stroke Unit - AOU, Ferrara, Italy; Università di Messina UOC Neuroradiologia, Messina, Italy; Stroke Unit -Policlinico “G.Martino,” Messina, Italy; UOC Neuroradiologia- AOU Consorziale Policlinico, Bari, Italy; UO Neurologia e Stroke Unit “F. Puca”- AOU Consorziale Policlinico, Bari, Italy; University of Turin, Torino, Italy; Stroke Unit – Molinette, Torino, Italy; SC Radiologia e Interventistica-AOU Alessandria, Italy; S.C. Neurologia e Stroke Unit, AOU Santi Antonio e Biagio, Alessandria, Italy; UO Neuroradiologia AOU Pisana, Italy; UO Neurologia, AOU Pisana, Pisa, Italy; Department of Neuroradiology, Hospital of Bolzano (SABES-ASDAA), Teaching Hospital of Paracelsus Medical University (PMU), Bolzano-Bozen, Italy; Department of Neurology, Hospital of Bolzano (SABES-ASDAA), Teaching Hospital of Paracelsus Medical University (PMU), Bolzano-Bozen, Italy; UOC Neuroradiologia Ospedale Vito Fazzi, Lecce, Italy; UOC Neurologia Ospedale Vito Fazzi, Lecce, Italy; UOC Neuroradiologia, Ospedale dell’Angelo, Mestre, Italy; UOC Neurologia, Ospedale dell’Angelo, Mestre, Italy; UOC Radiologia Diagnostica ed Interventistica, Ospedale SS Filippo e Nicola, Avezzano, Italy; UOC Neurologia e Stroke Unit, Ospedale SS Filippo e Nicola, Avezzano, Italy; IRCCS Istituto delle Scienze Neurologiche di Bologna, Neuroradiology Unit, Maggiore Hospital, Bologna, Italy; IRCCS Istituto delle Scienze Neurologiche di Bologna, Department of Neurology and Stroke Center, Maggiore Hospital, Bologna, Italy; Radiologia e Neuroradiologia Diagnostica e Interventistica, IRCCS Policlinico San Matteo, Pavia, Italy; UOC Neurologia d’Urgenza e Stroke Unit, IRCCS C. Mondino, Pavia, Italy; Unità Complessa di Neuroradiologia, Azienda Ospedaliero-Universitaria, Parma, Italy; Dipartimento di Medicina e Chirurgia, Università degli Studi di Parma, Parma, Italy; Programma Stroke Care, Dipartimento di Emergenza e Urgenza, Azienda Ospedaliero-Universitaria, Parma, Italy; SOC Radiologia Vascolare e Interventistica, Ospedale di Udine, Italy; University of Udine, Head, Neck and Neurosciences, Udine, Italy; UO Radiologia, A.O.O.R. Villa Sofia-Cervello, Palermo, Italy; UOC Neurologia con Stroke Unit, A.O.O.R. Villa Sofia-Cervello, Palermo, Italy; UOC Neuroradiologia, Rovigo, Italy; UOS Stroke Unit, Osp. Santa Maria Misericordia, Rovigo, Italy; UO Radiologia Interventistica, Azienda Ospedaliera “S. Maria,” Terni, Italy; Neurologia e Stroke Unit; Centro Cefalee, Dipartimento di Neuroscienze, Azienda Ospedaliera “S. Maria,” Terni, Italy; UO Neuroradiologia, Cremona, Italy; UOC Neurologia ASST, Cremona, Italy; UOC Neuroradiologia Diagnostica e Terapeutica AOU Senese, Italy; UOC Stroke Unit AOU Senese, Italy; UO Neuroradiologia, ARNAS Garibaldi CT, Catania, Italy; UO Neurologia ARNAS Garibaldi CT, Catania, Italy; SODC Interventistica Neurovascolare AOU Careggi, Firenze, Italy; Ospedale Mazzini, Teramo, Italy; Neurologia e Stroke Unit; Centro Cefalee, Azienda Ospedaliera “S. Maria,” Teramo, Italy; IRCCS Neuromed, Pozzilli (IS), Italy; Interventional Neuroradiology, Tor Vergata University, Rome, Italy; Interventional Neuroradiology, Sapienza University, Rome, Italy; Interventional Neuroradiology, S. Andrea Hospital, Rome, Italy; Emergency Department Stroke Unit, Department of Human Neurosciences, Sapienza University of Rome, Rome, Italy

**Keywords:** Acute ischemic stroke, intravenous thrombolysis, door-to-needle time, mechanical thrombectomy, outcome%

## Abstract

**Introduction:**

We aim to evaluate the association between door-to-needle time (DTN) and outcomes in a population of acute ischemic stroke (AIS) patients treated with intravenous thrombolysis (IVT) + mechanical thrombectomy (MT) in the Italian Registry of Endovascular Treatment in Acute Stroke (IRETAS).

**Materials and methods:**

Patients with AIS secondary to middle cerebral artery or intracranial internal carotid artery occlusion with known times of symptoms onset, directly presenting to an MT-capable center, were included in the analysis. According to pre-defined DTN cut-off values (⩽30, ⩽45, and ⩽60 min), we evaluated the association between DTN and outcomes by multivariate logistic regression analyses. Effectiveness outcomes were 3-month functional independence, 3-month excellent outcome and successful reperfusion. Safety outcomes were any intracranial hemorrhage (ICH), symptomatic intracerebral hemorrhage (sICH), and 3-month mortality.

**Results:**

About 1602 patients were included in our analysis. After logistic regression analysis, a DTN ⩽ 60 min was significantly associated with 3-month functional independence (OR 1.36; 95% CI 1.02–1.82). DTNs ⩽ 30, ⩽45, and ⩽60 min were significantly associated with successful reperfusion (OR 2.66; 95% CI 1.6–4.43; OR 1.68; 95%CI 1.25-2.26; OR 1.57; 95% CI 1.21–2.05; respectively). A DTN ⩽ 60 min was also significantly associated with lower rate of any ICH (OR 0.61; 95% CI 0.43–0.86). DTNs ⩽ 30, ⩽45, and ⩽60 min were significantly associated with lower 3-month mortality (OR 0.24; 95% CI 0.08–0.67; OR 0.45; 95% CI 0.29–0.72; OR 0.58; 95% CI 0.39–0.84; respectively).

**Conclusions:**

In patients with AIS treated with IVT + MT, a shorter DTN is associated with better outcomes if IVT is initiated within 1 h of hospital admission.

## Graphical Abstract

**Graphical abstract:**
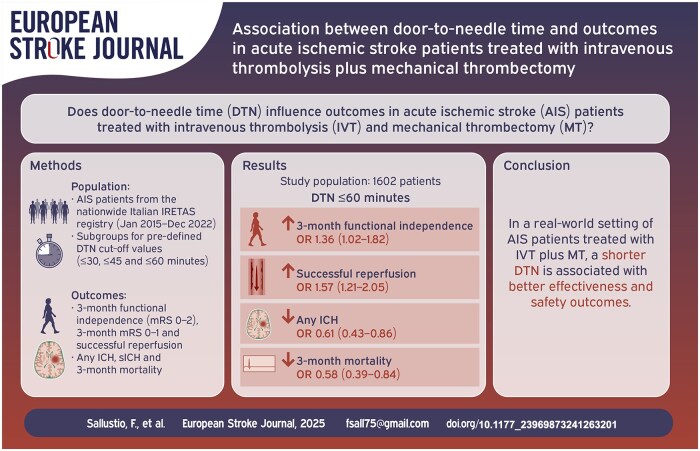

## Introduction

Randomized clinical trials (RCTs) comparing the efficacy and safety between intravenous thrombolytic therapy (IVT) plus mechanical thrombectomy (MT) and direct MT in acute ischemic stroke (AIS) patients have shown heterogeneous results.^1–6^ Despite this, an individual metanalysis of these trials investigating 2313 patients failed to show non-inferiority of direct MT compared to combined therapy.^7^ Guidelines recommend that patients with AIS secondary to large vessel occlusion (LVO) admitted to MT-capable centers should undergo IVT plus MT if eligible for both treatments.^8–10^

The above-mentioned trials did not demonstrate clear subgroup effects related to time from symptoms onset to randomization.^1–6^ A recent subanalysis of the randomized controlled SWIFT-DIRECT trial failed to show evidence that patients with a shorter onset-to-needle time (OTN) benefited more from IVT plus MT.^11^ Finally, a metanalysis of the six trials on the comparison between direct MT and combined therapy has shown that the treatment effect of IVT before MT is time-dependent.^12^

The aim of our study was to evaluate the association between door-to-needle time (DTN) and clinical outcomes in the real-world setting by investigating a large population of AIS patients treated with IVT + MT from the Italian Registry of Endovascular Treatment in Acute Stroke (IRETAS).^13^

## Methods

### Study population

This was a cohort study based on prospectively collected data of patients included in the IRETAS, a multicenter, observational, internet-based registry of patients with AIS due to LVO receiving endovascular treatment.^13^ Participating centers were required to include consecutive AIS patients receiving endovascular procedures. This study complies with the ethical standards laid down in the 1964 Declaration of Helsinki and its later amendments. Ethical approval was obtained by the Italian Ministry of Health and patient consent for participation in the IRETAS varied among participating hospitals according to local committees. Our analysis was conducted according to the STROBE criteria (Strengthening the Reporting of Observational Studies in Epidemiology) for observational studies.

We included patients with AIS secondary to anterior circulation LVO (middle cerebral artery or intracranial internal carotid artery) assessed by CT angiography scan, admitted to hospital according to a mothership paradigm or directly presenting to MT-capable centers, and treated with IVT + MT with an OTN ⩽ 4.5 h and an onset-to-groin time (OTG) ⩽ 8 h, between January 2015 and December 2022. We excluded patients with tandem or multiple vessel occlusions, unknown time of symptoms onset, wake-up and in-hospital AIS, unavailable 3-month functional status.

### Clinical, radiological, and procedural variables

The following clinical data were collected: demographic data such as age and sex; vascular risk factors such as hypertension, diabetes mellitus, dyslipidemia, atrial fibrillation, smoking status, previous stroke/transient ischemic attack (TIA), and coronary artery disease (CAD); pre-stroke modified Rankin Scale (mRS); pre-stroke cognitive impairment; baseline National Institutes of Health Stroke Scale (NIHSS); 24-h NIHSS.

Radiological and procedural data were: Alberta Stroke Program Early Computed Tomography Score [ASPECTS] on CT; site of occlusion; leptomeningeal collateral status, evaluated by means of the Careggi collateral score; symptoms onset-to-door time (OTD); OTN; symptoms onset-to-groin puncture time (OTG); symptoms onset-to-reperfusion time (OTR); door-to-CT time (DTC); DTN; door-to-groin puncture time (DTG); door-to-reperfusion time (DTR); procedural time (i.e. time from groin puncture to the reopening of the occluded vessel or, in case of failure, to the end of the procedure); general anesthesia during MT.

DTN for the entire cohort at each comparison point was categorized using predefined cut-offs (⩽30, ⩽45, and ⩽60 min). These align with American Heart Association/American Stroke Association (AHA/ASA) time targets^8,14^ and were chosen to facilitate interpretation of DTN’s impact on clinical outcomes.

### Outcome measures

Outcomes were defined as follows:

- procedural outcomes: (a) number of MT attempts; (b) rate of first pass recanalization; (c) intraprocedural complications such as vessel perforation, vessel dissection and/or distal embolization;
- effectiveness outcomes: (a) 3-month functional independence (defined as mRS ⩽ 2); (b) 3-month excellent outcome (defined as mRS ⩽ 1); (c) successful reperfusion (defined as TICI 2b-3);
- safety outcomes: (a) any intracranial hemorrhage (ICH; i.e. haemorrhagic infarction, parenchymal hematoma or subarachnoid hemorrhage) at 24-h imaging (or before, in case of clinical deterioration); (b) symptomatic ICH (sICH; defined as a parenchymal hematoma with an increase of ⩾4 NIHSS score points from baseline or death within 24 h) according to ECASS III criteria^15^; (c) 3-month mortality;

### Statistical analysis

Continuous variables were expressed as mean ± standard deviation (SD) or median with interquartile range (IQR), depending on the normality of their distribution. Categorical variables were expressed as count and percentages.

The Shapiro-Wilk test was used to assess the normality of continuous variables distribution. Appropriate statistical tests such as Mann–Whitney *U* test, Welch’s *t*-test and Fisher’s exact test or Chi-square test were used to examine differences in clinical, radiological, and procedural characteristics and outcomes between groups.

To better investigate in-hospital time metrics, we evaluated the relationship between DTN and DTG using Pearson correlation coefficient.

To evaluate the association between DTN and the probability of different outcomes we first performed univariate analyses.

Subsequently, multivariable logistic regression models were constructed. In these models DTN was initially treated as a continuous variable. To visualize the relationship between DTN and the probability of each outcome, linear regression plots were generated and the goodness of fit was assessed using coefficient of determination (*R*-squared).

Further, we performed multivariable analyses using predefined DTN cut-off values (⩽30, ⩽45, and ⩽60 min). Subdividing the entire cohort for each cut-off, logistic regression models were constructed to assess the association between DTN and outcomes. Variables with a *p*-value <0.1 in univariate analyses were considered candidates for inclusion in the models. Moreover, covariates were also selected with a multi-step, knowledge-informed approach, consistent with the principles of purposeful variable selection. Therefore, each model was adjusted for defined and known confounding factors, such as patient characteristics (age, sex), vascular risk factors (hypertension, diabetes mellitus, dyslipidemia, atrial fibrillation, smoking status), pre-stroke functional independence (pre-stroke mRS), and radiological and procedural characteristics (baseline NIHSS, ASPECTS on CT, OTG, successful reperfusion, any ICH). Confounding was evaluated using a change-in-estimate approach. Finally, multicollinearity was assessed in the final models by calculating Variance Inflation Factors (VIFs).

To further evaluate the relationship between DTN and the distribution of functional outcomes at 3 months, we performed an ordinal shift analysis for different DTN intervals (⩽30, 31–45, 46–60, and >60 min). To assess the statistical significance of any observed shift in mRS distribution between DTN subgroups, we used the Cochran-Mantel-Haenszel test.

Variables with more than 20% missing data were excluded from analyses according to the scientific rules of the IRETAS. To deal with missing data for the remaining variables we employed pairwise deletion.

All statistical analyses were performed using SPSS version 26 (IBM SPSS Statistics, Armonk, NY) and Microsoft Excel for Microsoft 365 (Version 2311 Build 16.0.16827.20198) 64-bit. Statistical significance was defined as a two-sided *p*-value < 0.05.

## Results

**Figure 1.**
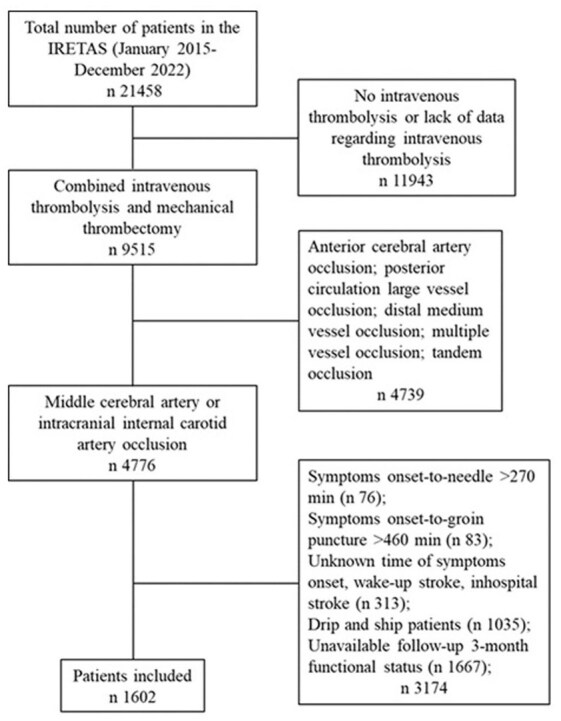
Flowchart of the study cohort.

Among 21,458 patients whose data were collected in the IRETAS between January 2015 and December 2022, 1602 were selected for analyses (Figure 1).

Mean age of the overall sample was 73.7 years (standard deviation ± 13.7) while the rate of male sex was 44.3%. Clinical, radiological and procedural variables are presented in Table 1. The rate of arterial hypertension and atrial fibrillation was significantly higher in the three longer DTN subgroups whereas the rate of pre-morbid absence of disability (i.e. mRS 0) was significantly higher in the three shorter DTN subgroups. All time metrics were significantly shorter in the three shorter DTN subgroups, except from OTD, which was longer in the shorter DTN subgroups.

**Table 1.** Clinical, radiological and procedural characteristics of study patients according to predefined door-to-needle cut-off groups (⩽30 min vs > 30 min; DTN ⩽ 45 min vs > 45 min; DTN ⩽ 60 min vs > 60 min).

Variable	Overall (n 1602)	DTN^a^ ⩽ 30 min (*n* = 178)	DTN^a^ > 30 min (*n* = 1424)	*p*-Value	DTN^a^ ⩽ 45 min (*n* = 514)	DTN^a^ > 45 min (*n* = 1088)	*p*-Value	DTN^a^ ⩽ 60 min (*n* = 870)	DTN^a^ > 60 min (*n* = 732)	*p*-Value
Age (years), mean (±SD^b^)	73.7 (13.7)	72.2 (14.3)	73.9 (13.6)	0.151	72.7 (14.1)	74.2 (13.5)	0.054	72.9 (14)	74.7 (13.3)	**0.009**
Sex (male), *n* (%)	710 (44.3)	86 (43.8)	124 (48.3)	0.255	233 (45.3)	477 (43.8)	0.576	399 (45.9)	331 (42.5)	0.175
Hypertension, *n* (%)	1067 (66.6) [22]	105 (59)	962 (68.6) [22]	**0.010**	314 (61.6) [4]	753 (70.4) [18]	**<0.0001**	555 (64.5) [10]	512 (71.1) [12]	**0.005**
Diabetes mellitus, *n* (%)	228 (14.2) [22]	22 (12.4)	206 (14.7) [22]	0.404	56 (11) [4]	172 (16.1) [18]	**0.007**	113 (13.1) [10]	115 (16) [12]	0.111
Dyslipidemia, *n* (%)	404 (25.2) [22]	38 (21.3)	366 (26.1) [22]	0.171	99 (19.9) [4]	305 (28.5) [18]	**<0.0001**	195 (22.7) [10]	209 (29) [12]	**0.004**
Atrial fibrillation, *n* (%)	448 (28) [22]	34 (19.1)	414 (29.5) [22]	**0.004**	115 (22.5) [4]	333 (31.1) [18]	**<0.0001**	199 (23.1) [10]	249 (34.6) [12]	**<0.0001**
Smoking status, *n* (%)	244 (15.2) [22]	38 (21.3)	206 (14.7) [22]	**0.021**	78 (15.3) [4]	166 (15.5) [18]	0.910	139 (16.2) [10]	105 (14.6) [12]	0.387
Previous stroke/TIA,^c^ *n* (%)	25 (1.6) [22]	2 [1.1)	23 (1.6) [22]	0.603	7 (1.4) [4]	18 (1.7) [18]	0.830	13 (1.5) [10]	12 (1.7) [12]	0.806
CAD,^d^ *n* (%)	148 (9.2) [22]	12 (6.7)	136 (9.7) [22]	0.202	43 (8.4) [4]	105 (9.8) [18]	0.378	78 (9.1) [10]	70 (9.7) [12]	0.658
Pre-stroke mRS^e^ 0, *n* (%)	1254 (78.3)	151 (84.8)	1103 (77.4)	**0.024**	419 (81.5)	835 (76.7)	**0.032**	698 (80.2)	556 (76)	**0.039**
Cognitive impairment, *n* (%)	19 (1.2) [22]	2 (1.1)	17 (1.2) [22]	0.918	4 (0.8) [4]	15 (1.4) [18]	0.336	8 (0.9) [10]	11 (1.5) [12]	0.278
Baseline NIHSS,^f^ median (IQR^g^)	15 (9) [24]	15 (9)	15 (9) [24]	0.953	15 (9) [6]	15 (9) [18]	0.848	15 (10) [12]	15 (10) [12]	0.709
24-h NIHSS,^f^ median (IQR^g^)	6 (11) [272]	5 (9) [29]	7 (11) [243]	**0.04**	5 (9) [91]	7 (12) [181]	**<0.0001**	6 (10) [154]	8 (12) [118]	**<0.0001**
ASPECTS^h^ on CT, median (IQR^g^)	10 (2) [232]	10 (2) [30]	10 (2) [202]	0.735	10 (2) [99]	10 (2) [133]	0.555	10 (2) [158]	10 (2) [74]	0.960
Site of occlusion										
MCA^i^, *n* (%)	1309 (81.7)	75 (5.3)	1349 (94.7)	0.413	484 (31.9)	1031 (68.1)	0.662	824 (54.4)	691 (45.6)	0.783
Symptoms onset-to-door time (min), median (IQR^g^)	75 (50)	80 (4)	75 (51)	**0.019**	76 (55)	75 (51)	**0.017**	76.5 (53)	74 (48)	**0.005**
Symptoms onset-to-needle time (min), median (IQR^g^)	149 (42.5)	117.9 (47.8)	152.9 (48.04)	**<0.0001**	124.2 (47.6)	160.7 (45.5)	**<0.0001**	130.2 (46.6)	171.3 (42.6)	**<0.0001**
Symptoms onset-to-groin puncture time (min), median (IQR^g^)	200.04 (62.6) [26]	178.1 (61.6) [3]	202.8 (62.3) [23]	**<0.0001**	179.2 (57.8) [10]	209.8 (62.5) [16]	**<0.0001**	183.7 (58.4) [17]	219.3 (62.03) [9]	**<0.0001**
Symptoms onset-to-reperfusion time (min), median (IQR^g^)	263.1 (74.8) [84]	236.6 (61.6) [14]	266.3 (74.4) [70]	**<0.0001**	239.4 (70.98) [37]	274 (74.03) [47]	**<0.0001**	244 (69.8) [57]	285.1 [27]	**<0.0001**
Door-to-CT time (min), median (IQR^g^)	33.7 (58.4) [51]	17.4 (10.97) [16]	35.6 (61.3) [35]	**<0.0001**	23.4 (71.2) [29]	38.3 (50.9) [22]	**0.002**	25.1 (55.9) [37]	43.6 (59.6) [14]	**<0.0001**
Door-to-groin puncture time (min), median (IQR^g^)	117.5 (48.8) [27]	87.3 (38.02) [3]	121.3 (48.6) [24]	**<0.0001**	90.3 (32.8) [10]	130.3 (49.8) [17]	**<0.0001**	97 (35.3) [17]	141.8 (51.3) [10]	**<0.0001**
Door-to-reperfusion time (min), median (IQR^g^)	180.3 (65.4) [85]	145.6 (58.4) [14]	184.5 (64.9) [71]	**<0.0001**	149.8 (52.7) [37]	194.3 (65.9) [48]	**<0.0001**	156.8 (53.9) [57]	207.5 (66.98) [28]	**<0.0001**
Procedural time (min), mean (±SD^b^)	62.6 (39.8) [73]	59.8 (37.6) [13]	65.9 (42.1) [60]	0.692	59.5 (37.2) [32]	64.1 (40.9) [41]	0.094	59.8 (37.6) [51]	65.9 (42.1) [22]	**0.013**
General anesthesia *n* (%)	583 (36.4)	82 (51.2)	501 (41.6)	**0.020**	206 (45.4)	377 (41.4)	0.160	331 (44.4)	252 (40.7)	0.174

Numbers in squared brackets indicate missing values. Significant *p*-values are in bold.

^a^Door-to-needle time.

^b^Standard deviation.

^c^Transient ischemic attack.

^d^Coronary artery disease.

^e^Modified Rankin Scale.

^f^National Institute of Health Stroke Scale.

^g^Interquartile range.

^h^Alberta Stroke Program Early CT Score.

^i^Middle cerebral artery.

We found a positive and statistically significant correlation between DTN and DTG (Pearson correlation coefficient *r* = 0.585, *p* < 0.001, *n* = 1575; Linear *R*² = 0.342).

Table 2 shows effectiveness and safety outcomes. The rate of 3-month functional independence, 3-month excellent outcome and successful reperfusion were significantly higher in the three shorter DTN subgroups. Among safety outcomes, the rate of any ICH was significantly lower in the DTN ⩽ 45 min and in the DTN ⩽ 60 min subgroups compared with the longer DTN counterparts. No significant difference in the rate of sICH was found between groups, whereas 3-month mortality was significantly lower in the three shorter DTN subgroups.

**Table 2.** Effectiveness and safety outcomes of study patients according to predefined door-to-needle cut-off values.

Variable	DTN^a^ ⩽ 30 min (*n* = 178)	DTN^a^ > 30 min (*n* = 1424)	*p*-Value	DTN^a^ ⩽ 45 min (*n* = 514)	DTN^a^ > 45 min (*n* = 1088)	*p*-Value	DTN^a^ ⩽ 60 min (*n* = 870)	DTN^a^ > 60 min (*n* = 732)	*p*-Value
Effectiveness outcomes									
3-Month functional independence, *n* (%)	113 (67.3) [10]	771 (57.2) [77]	**0.013**	310 (64.3) [32]	574 (55.6) [55]	**0.001**	518 (63) [48]	366 (52.8) [39]	**<0.0001**
3-Month excellent outcome, *n* (%)	91 (54.2) [10]	591 (43.9) [77]	**0.011**	247 (51.2) [32]	435 (42.1) [55]	**0.001**	401 (48.8) [48]	281 (40.5) [39]	**0.001**
Successful reperfusion, *n* (%)	145 (84.3) [6]	970 (70.3) [44]	**<0.0001**	390 (78.3) [16]	725 (68.1) [34]	**<0.0001**	629 (75.2) [34]	486 (67.9) [16]	**0.001**
Safety outcomes									
Any ICH,^b^ *n* (%)	38 (22.1) [6]	344 (24.4) [15]	0.502	100 (19.7) [7]	282 (26.3) [14]	**0.005**	173 (20.1) [8]	209 (29.1) [13]	**<0.0001**
sICH,^c^ *n* (%)	1 [13]	30 (2.2) [90]	0.244	8 (1.7) [31]	23 (2.3) [72]	0.440	13 (1.6) [49]	18 (2.7) [54]	0.147
3-Month mortality, *n* (%)	9 (5.4) [10]	215 (16) [77]	**<0.0001**	42 (8.7) [32]	182 (17.6) [55]	**<0.0001**	91 (11.1) [48]	133 (19.2) [39]	**<0.0001**

Numbers in squared brackets indicate missing values. Significant *p*-values are in bold.

^a^Door-to-needle time.

^b^Intracranial hemorrhage.

^c^Symptomatic intracerebral hemorrhage.

The following variables were excluded from analyses because of >20% missing data: leptomeningeal collateral status (55.8%), number of MT attempts (47.7%), rate of first pass recanalization (66.4%) and intraprocedural complications (47.7%).

**Figure 2.**
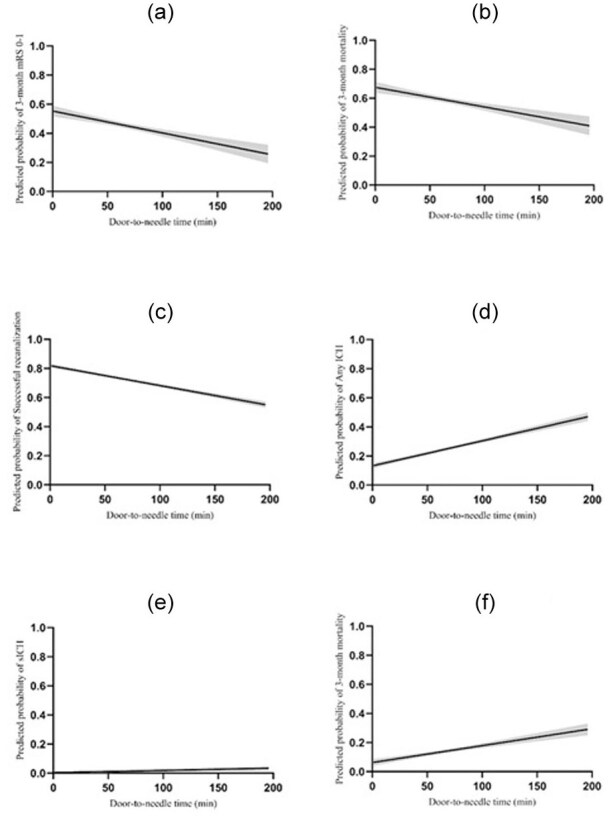
Adjusted linear regression plots of door-to-needle time and effectiveness and safety outcomes. The plots show the predicted probability of each outcome for different values of DTN. (a) 3-month excellent outcome, (b) 3-month functional independence, (c) successful reperfusion, (d) 3-month mortality, (e) any ICH, and (f) sICH. The models are adjusted for age, sex (male), hypertension, diabetes mellitus, dyslipidemia, atrial fibrillation, smoking status, pre-stroke mRS, baseline NIHSS, ASPECTS on CT, symptoms onset-to-door time, successful reperfusion, any intracranial hemorrhage, successful reperfusion. The shaded areas represent the 95% confidence intervals for the regression lines. The relatively narrow confidence intervals suggest that the estimates of the associations between DTN and outcomes are robust and reliable.

The association between DTN and effectiveness and safety outcomes is graphically represented by linear regression plots (Figure 2). An increase in DTN was associated with a reduced probability of 3-month excellent outcome, 3-month functional independence, and successful reperfusion. A shorter DTN was associated with a reduced probability of any ICH and a lower 3-month mortality. No association was found with sICH.

Table 3 shows unadjusted and adjusted logistic regression analyses for all effectiveness and safety outcomes. After adjusted logistic regression analyses, a DTN ⩽ 60 min was the only cut-off significantly associated with 3-month functional independence (OR 1.36; 95% CI 1.02–1.82), while all three DTN cut-offs were significantly associated with successful reperfusion (⩽30 min: OR 2.66; 95% CI 1.6–4.43; ⩽45 min: OR 1.68; 95% CI 1.25–2.26; ⩽60 min: OR 1.57; 95% CI 1.21–2.05). Regarding safety outcomes, DTN ⩽ 60 min was significantly associated with reduction of any ICH (OR 0.61; 95% CI 0.43–0.86), whereas the three shorter DTNs were significantly associated with reduction of 3-month mortality (⩽30 min: OR 0.24; 95% CI 0.08–0.67; ⩽45 min: OR 0.45; 95% CI 0.29–-0.72; ⩽60 min: OR 0.58; 95% CI 0.39–0.84). No association was found between DTN and sICH. The final multivariable models were checked for confounding and multicollinearity. No additional significant confounders were identified (change in the primary OR < 15% for all tested variables) and the models were free of significant multicollinearity (all VIFs < 1.5).

**Table 3.** Multivariate analysis of effectiveness and safety outcomes of study patients according to predefined door-to-needle cut-off values.

Variable	DTN^a^ ⩽ 30 min	*p*-Value	DTN^a^ ⩽ 45 min	*p*-Value	DTN^a^ ⩽ 60 min	*p*-Value
3-Month functional independence						
Unadjusted OR^b^ (95% CI^c^)	1.53 (1.09–2.16)	**0.013**	1.44 (1.15–1.8)	**0.001**	1.52 (1.2–1.87)	**<0.0001**
Adjusted,* OR^b^ (95% CI^c^)	1.34 (0.85–2.11)	0.212	1.14 (0.84–1.56)	0.399	1.36 (1.02–1.82)	**0.036**
3-Month excellent outcome						
Unadjusted OR^b^ (95% CI^c^)	1.51 (1.1–2.1)	**0.012**	1.45 (1.16–1.8)	**0.001**	1.4 (1.14–1.71)	**0.001**
Adjusted,* OR^b^ (95% CI^c^)	1.28 (0.83–1.96)	0.267	1.17 (0.87–1.58)	0.307	1.14 (0.86–1.51)	0.351
Successful reperfusion						
Unadjusted OR^b^ (95% CI^c^)	2.27 (1.48–3.48)	**<0.0001**	1.64 (1.27–2.1)	**<0.0001**	1.44 (1.15–1.8)	**0.001**
Adjusted° OR^b^ (95% CI^c^)	2.66 (1.6–4.43)	**<0.0001**	1.68 (1.25–2.26)	**0.001**	1.57 (1.21–2.05)	**0.001**
Any ICH						
Unadjusted OR^b^ (95% CI^c^)	0.88 (0.6–1.28)	0.502	0.69 (0.53–0.89)	**0.005**	0.61 (0.49–0.77)	**<0.0001**
Adjusted^§^ OR^b^ (95% CI^c^)	1.00 (0.64–1.56)	0.997	0.76 (0.55–1.04)	0.082	0.61 (0.43–0.86)	**0.001**
sICH						
Unadjusted OR^b^ (95% CI^c^)	0.27 (0.04–1.96)	0.193	0.73 (0.32–1.64)	0.442	0.59 (0.29–1.21)	0.151
Adjusted^§^ OR^b^ (95% CI^c^)	0.42 (0.05–3.21)	0.399	1.08 (0.43–2.72)	0.875	0.85 (0.37–1.95)	0.696
3-Month mortality						
Unadjusted OR^b^ (95% CI^c^)	0.3 (0.15–0.59)	**0.001**	0.45 (0.31–0.64)	**<0.0001**	0.52 (0.39–0.7)	**<0.0001**
Adjusted,* OR^b^ (95% CI^c^)	0.24 (0.08–0.67)	**0.006**	0.45 (0.29–0.72)	**0.001**	0.58 (0.39–0.84)	**0.005**

The table presents unadjusted and adjusted odds ratios (ORs) and 95% confidence intervals (CIs) for the association between different door-to-needle time (DTN) categories and outcomes. For each DTN category presented (e.g. DTN ⩽ 30 min), the reference group for the OR calculation is its respective complementary category (e.g. DTN > 30 min). This applies analogously to the DTN ⩽ 45 min (vs DTN > 45 min) and DTN ⩽ 60 min (vs DTN > 60 min) categories.

A *p*-value <0.05 was considered statistically significant. Significant *p*-values are in bold.

^a^Door-to-needle time.

^b^Odds ratio.

^c^95% confidence interval.

^*^Adjusted for age, sex (male), hypertension, diabetes mellitus, dyslipidemia, atrial fibrillation, smoking status, pre-stroke mRS, baseline NIHSS, ASPECTS on CT, symptoms onset-to-groin puncture time, successful reperfusion, any intracranial hemorrhage.

^°^Adjusted for age, sex (male), hypertension, diabetes mellitus, dyslipidemia, atrial fibrillation, smoking status, pre-stroke mRS, baseline NIHSS, ASPECTS on CT, symptoms onset-to-groin puncture time.

^§^Adjusted for age, sex (male), hypertension, diabetes mellitus, dyslipidemia, atrial fibrillation, smoking status, pre-stroke mRS, baseline NIHSS, ASPECTS on CT, symptoms onset-to-groin puncture time, successful reperfusion.

**Figure 3.**
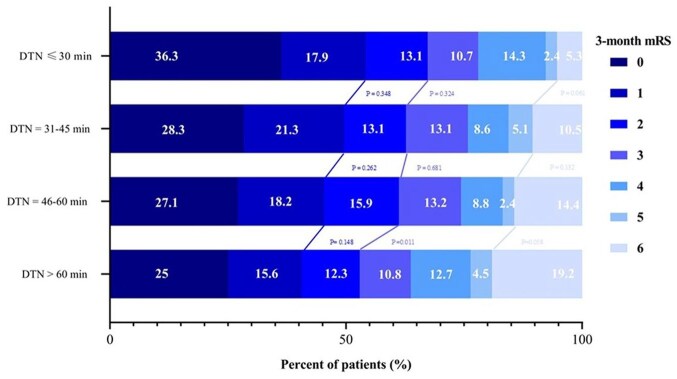
Ordinal shift analysis of 3-month modified Rankin Scale in predefined door-to-needle cut-off subgroups. Ordinal shift analysis of 3-month modified Rankin Scale (mRS) scores across mutually exclusive door-to-needle time (DTN) intervals. Each bar represents a specific DTN interval (⩽30 min, 31–45 min, 46–60 min, and >60 min). The segments within each bar indicate the percentage of patients within that interval achieving a specific mRS score (0 through 6) at 3 months, with color coding as per the legend. The figure visually demonstrates a shift in the mRS score distribution across these DTN intervals. A trend is observed whereby patients in shorter DTN intervals tend to exhibit better functional outcomes and lower mortality.

Figure 3 shows the ordinal shift analysis of the distribution of 3-month mRS scores across different DTN intervals, with a trend toward improved functional outcomes and reduced mortality with shorter DTNs.

## Discussion

In a real world setting of patients with AIS secondary to anterior circulation LVO, presenting directly to MT-capable centers and treated with IVT plus MT, a shorter DTN was associated with better effectiveness and safety outcomes. A benefit in terms of 3-month functional independence, successful reperfusion, hemorrhagic transformation and 3-month mortality was found when IVT was started within 1 h of hospital admission.

In the last few years, research and general attention amongst physicians have mostly focused on the comparison between combined treatment and direct MT to explore the benefit of IVT in MT patients.^1–6^ It is well-known from RCTs and large retrospective studies based on registries, that the benefit of intravenous recombinant tissue-type plasminogen activator (rtPA) in AIS is time dependent.^16,17^ Some of the previous studies exploring this issue used indirect measures of motor recovery to define functional outcome, such as ambulatory status at discharge or discharge destination instead of a thorough evaluation of mRS.^17^ Similarly to our findings, the HERMES collaboration found that shorter DTNs are associated with better outcomes in patients treated with MT but this study included only 601 highly selected trial patients potentially not representative of the routine clinical practice.^18^

Recent studies have tried to answer the question of time-dependent effect of IVT prior to MT. A large nationwide US Get-with-the-Guidelines registry showed that shorter DTNs were associated with better clinical outcomes. The main outcome measure explored was the home time at 90 days which showed to be increased with shorter DTNs, with a parallel decrease in mortality and an increase in odds of zero home time for each 15-min increase in DTN. No analysis of 3-month mRS was reported.^19^

Interestingly, our analysis shows that older age, hypertension, atrial fibrillation and pre-stroke disability are more common in patients treated with more delay. This could be due to the longer time needed to assess eligibility for treatment in patients with multiple risk factors. Moreover, the time needed to arrive to the hospital from the onset scenario was significantly longer in the three subgroups treated with shorter DTN as compared with their longer counterparts, which may be due the physician’s effort to compensate the pre-hospital delays by reducing intrahospital time metrics.^20^

The groups were well balanced in terms of other baseline characteristics such as NIHSS, ASPECTS, previous stroke or TIA or cognitive impairment, suggesting a rigorous process of patient selection for reperfusion therapies since these are well-recognized prognostic factors of outcome in the stroke population.^21,22^ In this regard, tandem occlusions or other multiple vessel occlusions were not included in the analysis to mitigate potential bias secondary to neuro-interventionalists’ experience and skills in treating more technically complex occlusions.

Some interesting procedural outcomes such as number of recanalization attempts during MT and first pass recanalization effect could not be described because of missing data. However, patients with a DTN ⩽ 60 min showed a significantly shorter procedural time compared to those with a DTN > 60 min. Despite the proven benefit of recanalization, longer procedures and more MT attempts have shown to be detrimental in both successful and unsuccessful procedures.^23^ Furthermore, a recent study from our registry demonstrated how procedural time might be associated with outcomes in patients with unsuccessful recanalization regardless of the number of MT attempts.^24^ Therefore, earlier treatment with IVT might also facilitate faster achievement of successful reperfusion leading to a shorter procedural time. In fact, over time the thrombus becomes denser and more organized, and subsequently more difficult to remove with reperfusion therapies.^25^

In our study, earlier treatment with IVT at any cut-off value was independently associated with reduced mortality, which is consistent with previous retrospective studies.^18^ While no association with sICH was found, shorter DTNs were associated with less hemorrhagic transformation (any ICH). A possible explanation is that, as successful reperfusion rate increases with shorter DTNs, it is likely that a faster treatment with IVT will lead to a smaller size of infarction, probably reducing the risk of hemorrhagic transformation. This is of relevance when considering that the occurrence of any hemorrhagic complication after reperfusion therapies might affect stroke physicians’ decision on the timing of initiation of secondary prevention and that discontinuation of the latter could increase recurrent stroke and mortality.^26^

Of note, only around 55% of patients in our population achieved a DTN of less than 1 h, a lower percentage than the recommended goal on timely treatment with IVT from the American Heart Association.^27^ This should raise awareness about the continuous need to implement stroke treatment pathways in our network. Moreover, this finding may have been the cause of the lack of association between 3-month functional independence and lower DTN cut-offs because of the reduced sample size of these groups of patients.

Our findings provide supporting evidence that IVT should be commenced as soon as possible after non-contrast CT scan.^8^ In our work, we decided to focus on the in-hospital phase of AIS management, which is a useful target of initiatives aiming at ameliorating patients’ care.^27^ However, efforts should be made to avoid any delays in the processes preceding the administration of IVT both in the in-hospital and pre-hospital settings. Mobile stroke units, which are set up to deliver thrombolytic treatment in the pre-hospital setting, might represent the best option to achieve the goal of ultra-early IVT administration in acute stroke patients, possibly within 60 min of symptoms onset (the so-called golden hour).^28^ However, its availability is limited in most countries and therefore, in this scenario, administering IVT as soon as possible after hospital admission remains of paramount importance.

As expected, in our population we found a moderate positive correlation between DTN and DTG. In fact, in clinical practice, a delay in IVT administration is usually also reflected in the timing of subsequent endovascular therapy. However, in our study DTN explained only approximately 34.2% of the variance in DTG, suggesting that DTN, while being a contributing factor to DTG, is not its sole determinant. Indeed, several critical in-hospital processes significantly contribute to the overall DTG, such as angiography suite preparation and the immediate availability of a dedicated neurointerventional team.

This study has some limitations. First, the retrospective nature of our analysis reduces the precision of our results. However, baseline characteristics were well balanced between DTN groups and in-hospital time metrics such as DTN time were prospectively collected in our registry and not indirectly derived from other time metrics. Second, the study only included patients with single vessel occlusion presenting directly to MT-capable centers, receiving IVT within 4.5 h and MT within 8 h of onset. Third, we cannot exclude potential bias in the accuracy of baseline and post-treatment imaging variables due to the absence of a core imaging lab for central imaging assessment. Fourth, all patients were treated with rtPA, so the results cannot be generalizable to IVT with tenecteplase. Fifth, the exclusion from analyses of those variables with more than 20% missing data might have affected the interpretation of the results. However, none of the key variables and none of the effectiveness and safety outcomes was excluded because of missing values, similarly to other nationwide stroke registries.^29^ Sixth, the multicenter collection of data in this registry implies heterogeneity in treatment protocols, an intrinsic bias to multisite registries that might have led to reveal only very robust statistical associations. Despite these limitations our study represents a solid analysis from a real world setting of a large nationwide registry, exploring the association between DTN and functional outcomes in MT patients treated with IVT. This study provides further evidence in support of the benefit of early treatment with IVT in LVO patients. Efforts to commence IVT as soon as possible should be made in all eligible patients, including LVO patients presenting to MT-capable centers.

## Conclusions

In anterior circulation LVO patients presenting to MT-capable centers treated with IVT plus MT, a shorter DTN is associated with higher rates of functional independence and successful reperfusion, and lower rates of mortality and hemorrhagic transformation. IVT in LVO patients presenting directly to MT-capable centers should not be delayed because of the planned thrombectomy.

## Data Availability

Anonymized data will be shared by reasonable request from any qualified investigator.

## ORCID iDs

Fabrizio Sallustio   https://orcid.org/0000-0002-8229-465X

Federico Marrama   https://orcid.org/0000-0003-2786-8312

Ilaria Casetta   https://orcid.org/0000-0003-4099-8875

Ettore Nicolini   https://orcid.org/0000-0002-8481-6327

Antonio Ciacciarelli   https://orcid.org/0000-0003-3282-4672

Alessandro De Vito   https://orcid.org/0000-0002-7572-819X

Mauro Bergui   https://orcid.org/0000-0002-5336-695X

Alessio Comai   https://orcid.org/0000-0002-0566-395X

Nicola Cavasin   https://orcid.org/0000-0001-6246-3543

Marco Perri   https://orcid.org/0000-0001-6113-262X

Giovanni Merlino   https://orcid.org/0000-0002-6525-9529

Marina Mannino   https://orcid.org/0000-0001-7683-7235

Stefano Caproni   https://orcid.org/0000-0002-8627-9879

Rossana Tassi   https://orcid.org/0000-0002-5906-8718

Edoardo Puglielli   https://orcid.org/0000-0002-3082-249X

Salvatore Mangiafico   https://orcid.org/0000-0002-5844-3117
